# Virulence of Legionella pneumophila strains isolated from hospital water system and healthcare-associated Legionnaires’ disease in Northern Italy between 2004 and 2009

**DOI:** 10.1186/1471-2334-14-483

**Published:** 2014-09-05

**Authors:** Savina Ditommaso, Monica Giacomuzzi, Susan R Arauco Rivera, Roberto Raso, Pierangela Ferrero, Carla M Zotti

**Affiliations:** Dipartimento di Scienze della Sanità Pubblica e Pediatriche, Università degli Studi di Torino, Via Santena 5 bis, 10126 Torino, Italy; Servizio di riferimento Regionale di Epidemiologia per la sorveglianza, la prevenzione e il controllo delle Malattie Infettive, SeREMI Alessandria, Alessandria, Italy

## Abstract

**Background:**

Worldwide, *L. pneumophila* sg 1 is the most common agent of Legionnaires’ disease ( 80 to 90% of the reported cases). In contrast, *L. pneumophila* sg 2–14 account for only 15 to 20% of community-acquired cases, although they account for over 50% of the environmental isolates. The discrepancy between environmental isolates and clinical cases of disease suggested that there are differences in virulence.

We decided to subtype the environmental *Legionella* strains isolated from health care facilities (HCFs) and to compare the distribution of strains with the occurrence of hospital-acquired legionellosis.

**Methods:**

Observational ecological study based on the data provided by the regional surveillance of legionellosis and on data obtained from hospitals environmental monitoring.

Using the monoclonal antibody MAb 3/1 of the Dresden Panel we collected and typed environmental strains of *L. pneumophila* sg 1 obtained during routine testing in 56 health care facilities from 2004 to 2009.

The results of the laboratory analyses of the environmental samples were compared with the number of cases that each health care facility reported during the study period.

**Results:**

The association between the type of colonisation (*L. pneumophila* sg 1 vs others serogroups) and the incidence of reported cases was statistically significant (p = 0.03 according to the χ^2^ test).

*Legionella* strains with the virulence–associated epitope recognised by MAb 3/1 were isolated in 8 of the 26 HCFs colonised by *L. pneumophila* sg 1; 7 of the HCFs colonised by MAb 3/1-positive strains accounted for 85% of the cases of hospital-acquired legionellosis reported during the 6-year study period. There was a statistically significant association (p = 0.003) between the presence of cases and colonisation by MAb 3/1-positive *Legionella* strains.

**Conclusion:**

This study suggests that hospitals colonised by more virulent strains should be aware of the increased risk and consider the opportunities of increase their monitoring efforts and implement more effective contamination control strategies.

## Background

Hospital water systems have been identified as a source of *Legionella* pneumonia. *Legionella pneumophila* serogroup 1 is the most common cause of legionellosis, a sporadic and endemic disease that may be acquired from different environmental sources [1].

The utility of environmental monitoring for *Legionella* species remains controversial.

Two different strategies for preventing hospital-acquired *Legionella* pneumonia have been advocated. One strategy emphasises environmental monitoring for *Legionella* species [2], and because the contamination of a hospital’s water supply by *Legionella* species can place inpatients at risk of developing hospital-acquired *Legionella* pneumonia, this strategy encourages pneumonia surveillance through diagnostic testing. An alternative strategy proposed by the United States Centers for Disease Control and Prevention [3] advocates intensive clinical surveillance without routine environmental surveillance, except in transplant units [4]. National and international guidelines for *Legionella* prevention and control set risk and intervention threshold levels for water distribution systems based on the *Legionella* load detected in samples. Currently, the French guidelines [5] are alone in recommending that risk levels be graded according to the quantity of microorganisms plus the *Legionella* species and serogroups present in a hospital’s water supply. Previously, we reported [6] the findings of a two-year prospective study on the incidence of nosocomial legionellosis in hospitals contaminated with *L. pneumophila* other than serogroup 1 or non*-Legionella pneumophila* species. In the hospitals that only performed regular ordinary maintenance without carrying out decontamination measures, the results (32 hospitals, 325,703 patients, only one case of healthcare-associated Legionnaires’ disease reported) confirm that the presence of *Legionella* in a hospital’s water distribution system does not necessarily lead to legionellosis [7, 8], and in hospitals contaminated with *L. pneumophila* non-sg 1, the risk of developing legionellosis is very low.

Worldwide, *L. pneumophila* sg 1 is the most common agent of Legionnaires’ disease, accounting for approximately 80 to 90% of the reported cases [9–11] and approximately 70% of European travel-associated cases [12]. In contrast, *L. pneumophila* sg 2–14 account for only 15 to 20% of community-acquired cases, although they account for over 50% of the isolates obtained from man-made aquatic systems. The discrepancy between environmental isolates and clinical cases of disease has been observed by Doleans et al. [13], who suggested that there are differences in virulence rather than greater abundance in water distribution systems. This discrepancy was also reported by Harrison et al. [14], who found a higher proportion of only a few restriction-fragment-length polymorphism (RFLP) types in clinical isolates compared with the more even distribution of RFLP types seen in environmental isolates.

In Italy, legionellosis is subject to special surveillance [15]. Physicians who diagnose legionellosis cases, whether they are hospital or community acquired, notify the local health authority via normal reporting channels. This information is then sent to the regional authorities (Regional Epidemiological Services for the Monitoring, Prevention and Control of Infectious Diseases [SeREMI), which then forward the information to the Ministry of Health, the Italian National Institute of Statistics (ISTAT) and the Istituto Superiore di Sanità (ISS). In the last 10 years, 164 hospital-acquired legionellosis cases occurring in Piemonte have been reported through this surveillance network; 112 of these cases were reported at two of the region’s major hospitals. This marked difference in the attack rates suggests that the *L. pneumophila* sg 1 strains in some hospitals may be more pathogenic than those circulating in hospitals where no cases of legionellosis have been reported. Therefore, we decided to subtype the *Legionella* strains isolated during environmental monitoring with monoclonal antibody MAb 3/1 of the Dresden Panel (corresponding to MAb 2 of the International Panel) directed against lipopolysaccharide epitopes on the surface of *Legionella* cells [16, 17]. According to epidemiological studies, this epitope appears to be associated with virulence [18, 19]. One possible explanation for the higher virulence potential of strains carrying the epitope recognised by MAb 3/1 is that legionellae are more likely to survive in aerosols, increasing the dose inhaled by exposed individuals. Immunochemical analysis of the epitope has suggested that the increased hydrophobicity of these strains encourages the survival of these legionellae in aerosols [20]. Gosselin et al. [21] studied the electrokinetic patterns of strains carrying the MAb3/1 epitope and provided a physicochemical explanation for the role played by the MAb3/1 epitope in Legionella cases. They demonstrated that virulent MAb3/1-positive strains have an average flow penetration length (1/λ_0_) that is 1.5 times larger and a volume charge density (ρ_0_) that is approximately 5 times lower than those derived from less virulent MAb3/1-negative strains.

The infection potential of *Legionella pneumophila* sg1 strains is most pronounced for bacteria with a low ρ_0_ and a large λ_0_ that carry the MAb3/1 epitope. The larger ρ_0_ is the more important the repulsion between *Legionella pneumophila* sg1 and host cell is, and the lower is the probability of these cells to be infected.

The purpose of this study was to compare the distribution of *L. pneumophila* sg 1 monoclonal subtypes obtained during routine sampling in HCFs water systems with the distribution of hospital-acquired legionellosis, to assess the risk associated with contaminated environmental reservoirs colonised by more virulent strains.

## Methods

### Study design

This is an observational ecological study based on the data provided by the regional surveillance of legionellosis and on data obtained from hospitals environmental monitoring of *Legionella.*

The number of cases that each health care facility reported was compared to the environmental strains of *Legionella* (detected, enumerated and serotyped)*.*

### Health care facilities (HCFs)

Altogether 56 HCFs were monitored, 46 were hospitals and 10 long term care facilities(LTCF).

The hospitals varied in size: 1250 beds (one hospital), 500–1000 beds (3 hospitals), 200–500 beds (15 hospitals) and <200 beds (27 hospitals). The mean age of the buildings was 40 years, but over the years, the water systems had been partially renovated.

Over the years, we have gained a fairly complete picture of the situation at 56 health care facilities (HCFs), contaminated with *Legionella*, that implement routine testing and management interventions. Until 2008, the majority of HCFs carried out regular controls twice a year (on a total of 20 samples per year). Since April of 2008, the Piemonte region authorities [22, 23] have invited HCF to implement environmental monitoring programmes that require quarterly sampling of their water supply and recirculation loops of boilers; this regime identifies fluctuations in the colonisation of *Legionella*. Sampling at distal sites is limited to the facilities where patients at high risk of infection are hospitalised. In addition to environmental monitoring, the Piemonte Regional authorities advocate clinical surveillance performed by the Infection Control Committee, emphasising that physicians should require *Legionella* diagnostic tests for patients with pneumonia.

In the first few years of environmental monitoring, all the hospitals proved to be contaminated by *Legionella spp*., although marked differences were seen in terms of microbial load and types of strains; the most frequently isolated strains were *L. pneumophila* serogroups 1, 3, 6. The age and size of the buildings were not associated with the degree of contamination. The massive contamination found during the first years of monitoring gradually diminished as a result of remedial intervention on the water systems monitored [24].

The hospitals were classified according to the Case-Mix Index (CMI), which was obtained from the Regional Department of Health (RDH). To calculate the CMI, RDH used the Medicare Severity-Diagnosis Related Group (MS-DRG) weights by averaging the MS-DRG weight of all patients discharged within the calendar. CMI describes the complexity of cases managed by every hospital in relation to the average complexity of Italian hospitals. In this study we used CMI as a confounding variable and considered the hospitals with a CMI greater than 1 as facilities with case - complexity higher than reference, as defined by the Ministry of health.

Long-term care facilities (LTCFs) are establishments where non-self-sufficient patients who cannot be assisted at home and can be accommodated for an indefinite period of time. The majority of patients in these establishments are elderly, with chronic morbidities and often confined to bed, conditions that are important risk factors for infection with Legionella spp. The sizes of these establishments varied from 30 beds to 177 beds. For the LTCF the case-mix index was not provided, so we arbitrarily assigned a value index < 1.

### Diagnosis of Legionellosis

Hospitals performed active surveillance for *Legionella* infection for all cases of nosocomial pneumonia identified by clinical, radiological, and laboratory criteria according to national and international case definition [3, 15].

In our setting, if a patient had pneumonia urine specimens were collected and examined for *Legionella* antigen; if the results were negative, the test was repeated 5–7 days later. Culture of respiratory secretions when possible was recommended, as was testing for specific antibodies on the onset of symptoms and after 15–20 days. Urinary antigen detection is sufficient to start a therapy and initiate an environmental investigation. The discovery of *Legionella* in the water supply led to initiate the disinfection of the plumbing system.

The following definition integrate the clinical and microbiological criteria that classify a patient as a nosocomial case of Legionnaires’ disease and is used for surveillance purposes:

“definite nosocomial” case of Legionnaires’ disease who was in a hospital or nursing home or other healthcare facility for at least ten days prior to the onset of symptoms.

The management of active surveillance was assigned to the hospital’s Infection Control Committee that, in collaboration with the infection control nurse, collected data about patients with nosocomial pneumonia and reported this information to the regional surveillance network (SeREMI). During the study period (2004–2009) this network had reported cases of hospital-acquired legionellosis that were detected by positive urine antigen tests; in any cases was available culture of respiratory secretions and species/serogroup information.

This study did not require ethical approval, we only accessed the number of cases that each health care facility referred to SeREMI and analyzed virulence only on environmental samples.

### Environmental isolates

We collected data (about 5600 water samples) and environmental strains obtained from routine testing conducted in 56 HCFs from 2004 to 2009. The water-testing data and environmental strains for 15 hospitals were furnished by the Regional Agency for Environmental Protection (ARPA Piemonte).

*Legionella spp*. were isolated using a cultural method according to the ISO 11731 standard technique [25].

Using MAbs 3/1, we typed the 264 environmental strains collected from the HCFs water supply colonised by *L. pneumophila* sg 1. We tested more than one strain from different years at each HCF to identify possible variations in the reactivity of MAb3/1, as suggested by Bernarder et al. [26].

### Monoclonal Subgrouping of isolates

The *L. pneumophila* sg 1 strains were classified according to whether they expressed the virulence-associated epitope (MAb 3/1-positive) or not (MAb 3/1-negative). The *L. pneumophila* sg 1 strains were typed with MAbs 3/1 of the Dresden panel using an indirect immunofluorescence test (IIFT) [16, 17]. *Legionella* bacteria were scraped from BCYE agar, fixed for 10 min in acetone, resuspended in distilled water to a concentration of approximately 3x10^8^ and subjected to indirect immunofluorescence testing [27]. The MAb 3/1 were used as undiluted cell culture supernatants, and goat antimouse fluorescein isothiocyanate conjugate (polyvalent) (Sigma Aldrich, St Louis, USA) was used after being diluted 1:64.

### Statistical analysis

The χ^2^ test, Fisher’s exact test and stratification by CMI were applied to determine whether there was an association between legionellosis cases and the type of colonisation in the health care facility’s water supply system. For data processing the case-mix index were grouped into two categories: hospitals with CMI < 1 and hospitals with CMI > 1.

## Results

Table 1 shows the main information (CMI, bed-sites, number of nosocomial legionellosis cases, colonisation by *Legionella* in water system) about the hospitals and the LCTFs.

**Table 1:** **Distribution of** ***Legionella*** **and nosocomial Legionnaires’ disease among Piemonte health care facilities (2004–2009)**

Health care facility code	Case mix	Number of beds	Nosocomial legionellosis cases	Legionella spp	MAb 3/1
1	1.62	1250	34	Lp1, Lp2-14, L.spp	+
2	1.1	732	3	Lp1, Lp2-14, L.spp,	+
3	1.32	559	17	Lp1, Lp2-14, L.spp,	+
4	0.81	289	8	Lp1, Lp2-14, L.spp,	+
5	1.06	203	9	Lp1	+
6	1.32	368	4	Lp1, L.spp	-
7	1.46	339	1	Lp1, Lp2-14, L.spp	-
8	1.22	722	1	Lp1, Lp2-14, Lspp	-
9	0.8	64	0	Lp1, Lspp	+
10	1.41	120	0	Lp1, L.spp	-
11	0.65	92	0	Lp1, L.spp	-
12	0.73	116	0	Lp1, Lp2-14, L.spp	-
13	0.9	65	0	Lp1, Lp2-14, Lspp	-
14	0.89	240	0	Lp1, L.spp	-
15	0.48	73	0	Lp1, L.spp	-
16	0.71	111	0	Lp1, Lp2-14, Lspp	-
17	0.88	255	0	Lp 1	-
18	1.26	412	0	Lp1, Lp2-14, Lspp	-
19	0.45	317	0	Lp1, Lp2-14, Lspp	-
20	1.02	108	0	Lp1, Lp2-14, Lspp	-
21	1.03	70	0	Lp1, Lp2-14, Lspp	-
22	0.83	160	1	Lp2-14	n.a
23	0.77	225	1	Lp2-14	n.a.
24	1.12	193	1	Lp2-14, L.spp	n.a.
25	1.27	448	1	Lp2-14	n.a.
26	0.92	482	2	Lp2-14, Lspp,	n.a
27	0.76	196	0	Lp2-14, Lspp,	n.a.
28	0.85	268	0	Lp2-14, Lspp	n.a.
29	0.78	57	0	Lp2-14	n.a
30	0.80	86	0	Lp2-14, Lspp,	n.a.
31	0.81	90	0	Lp2-14, Lspp	n.a.
32	0.80	30	0	Lp2-14, Lspp	n.a
33	0.82	54	0	Lp2-14, Lspp	n.a.
34	0.89	228	0	Lp2-14	n.a.
35	1.19	138	0	Lp2-14	n.a
36	0.93	85	0	Lp2-14 Lspp	n.a.
37	0.53	175	0	Lp2-14, Lspp	n.a.
38	0.90	242	0	Lp2-14	n.a
39	0.76	72	0	Lp2-14	n.a.
40	0.82	85	0	Lp2-14, Lspp	n.a.
41	0.48	482	0	Lp2-14, Lspp	n.a.
42	0.76	49	0	Lspp	n.a.
43	0.66	73	0	Lp2-14	n.a.
44	1.17	82	0	Lp2-14, Lspp	n.a
45	0.87	48	0	Lp3	n.a.
46	0.70	139	0	Lp2-14,Lspp	n.a.
47	/	110	2	Lp1, Lp2-14, L.spp	+
48	/	126	1	Lp1	+
49	/	117	1	Lp1, L.spp	-
50	/	174	0	Lp1,Lspp	-
51	/	60	0	Lp1	-
52	/	30	0	Lspp	n.a
53	/	60	0	Lp2-14	n.a.
54	/	174	0	Lp2-14, Lspp	n.a
55	/	60	0	Lp2-14	n.a.
56	/	177	0	Lspp	n.a

n.a. = not applicable.

Lp1 = *L. pneumophila* sg 1; Lp2-14 = *L. pneumophila* sg 2–14; L. spp = *L. species* non-*pneumophila.*

Based on the analysis of the strains isolated during environmental monitoring in the study period, the HCFs were classified as 1) facilities contaminated by *L. pneumophila* sg 1 alone or in association with other *Legionella* serogroups or species (26/56) or 2) as facilities contaminated by one or more *L. pneumophila* serogroups (sg 2–14) alone or in association with other *Legionella* species (30/56).

A total of 87 cases of hospital-acquired legionellosis were reported to the regional surveillance system (SeREMI) during the study period; the notifications originated from 16 of the 56 HCFs monitored (Table 1). Comparing the environmental data with the number of hospital-acquired legionellosis it emerges that 2 HCFs colonised with *L. pneumophila* sg 1 alone reported 10 cases; 5 HCFs that were not colonised with *L. pneumophila* sg 1 reported 6 cases, the remaining 9 HCFs colonised with several serogroups (*L. pneumophila* sg 1 and others serogroups) reported 71 cases.

The association between the type of colonisation (*L. pneumophila* sg 1 vs others serogroups) and the incidence of reported cases was statistically significant (p = 0.03 according to the χ^2^ test) (Table 2). Since diagnosis of *Legionella* infection was made by urinary antigen, linkage between infection and environment can only be hypothesized; in fact in 9 hospitals there was a colonization with several serogroups.

**Table 2:** **Association between colonisation with** ***L. pneumophila*** **sg 1 and reported cases of legionellosis**

	HCFs with reported cases	HCFs without reported cases	Total
HCFs colonised with *L. pneumophila* sg 1 and other serogroup and species	11	15	26
HCFs colonised with *L. pneumophila* sg 2–14 and other species	5	25	30
Total	16	40	56

p = 0.03 according to the χ^2^ test.

*Legionella* strains with the virulence–associated epitope recognised by MAb 3/1 were isolated in 8 of the 26 HCFs colonised by *L. pneumophila* sg 1; 7 of the HCFs colonised by MAb 3/1-positive strains accounted for 85% of the cases of hospital-acquired legionellosis reported during the 6-year study period.

Analysis of isolates during the years of the study showed variation of reactivity of MAb 3/1only in Hospital No. 9, where it was also isolated a strain 3/1 MAb negative.

There was a statistically significant association (p = 0.003 according to Fisher’s exact test) between the presence of cases and colonisation by MAb 3/1-positive *Legionella* strains (Table 3). One HCF (No. 9) had no cases of legionellosis even though it was colonised by MAb 3/1-positive *Legionella* strains. In contrast, one HCF (No. 6) reported 4 cases even though it was colonised by MAb 3/1-negative *Legionella*.

**Table 3:** **Legionellosis cases and colonisation with Legionella MAb 3/1 positive strains**

	HCFs with reported cases	HCFs without reported cases	Total
HCFs colonised with *L. pneumophila* sg 1 MAb 3/1 positive	7	1	8
HCFs colonised with *L. pneumophila* sg 1 MAb 3/1 negative	4	14	18
Total	11	15	26

p = 0.003 according to Fisher’s exact test.

No statistically significant differences (p = 0.7 according to Fisher’s exact test) were found in the occurrence of legionellosis for the HCFs colonised by MAb 3/1-negative *L. pneumophila* sg 1 compared with those colonised by *L. pneumophila* sg 2–14 (Table 4). This result suggests that there is no greater risk of infection HCFs in facilities colonised by *L. pneumophila* sg 1 without the virulence–associated epitope recognised by MAb 3/1 compared with those colonised by *L. pneumophila* sg 2–14.

**Table 4:** **Legionellosis cases in health care facilities colonised with** ***L. pneumophila*** **sg 2–14 and/or** ***L. pneumophila*** **sg 1 MAb 3/1 negative**

	HCFs with reported cases	HCFs without reported cases	Total
HCFs colonised with *L. pneumophila* sg 1 MAb 3/1 negative	4	14	18
HCFs colonised with *L. pneumophila* sg 2-14	5	25	30
Total	9	39	48

p = 0.7 according to Fisher’s exact test.

Furthermore, we analysed the 56 HCFs to determine whether the complexity of care (i.e. the case-mix) was also an important factor in the occurrence of legionellosis (Table 5). To this aim we merged the structures colonized by MAb 3/1-negative *L. pneumophila* sg 1 with those colonized by *L. pneumophila* sg 2–14. Analysis of the raw data (univariate analysis) showed an association between the presence of MAb and the presence of cases (p = 0.0006 by Fisher exact test). With the layering by a stratifying of the data we found a statistically significant association between colonisation and reported cases only in the HCFs classified as CMI < 1 (p = 0.02 according to Fisher’s exact test).

**Table 5:** **Association of MAb 3/1, CMI and reported cases**

	a) HCF		b) HCF with CMI < 1		c) HCF with CMI >1	
MAb 3/1	HCFs without reported cases	HCFs with reported cases	HCFs without reported cases	HCFs with reported cases	HCFs without reported cases	HCFs with reported cases
Negative(^a^)	39	9	33	4	6	5
Positive	1	7	1	3	0	4
	p = 0.0006		p = 0.02		p = 0.18	
	(Fisher’s exact test)		(Fisher’s exact test)		(Fisher’s exact test)	

(^a^) both the HCF colonized by L. pneumophila. sg 1 MAb 3/1 negative, and all other structures colonized by L. pneumophila sg 2–14.

## Discussion

*L. pneumophila* sg 1 is responsible for the majority of hospital-acquired infections. Some strains of the *L. pneumophila* sg 1 population have an enhanced ability to cause disease: the clinical predominance of *L. pneumophila* sg 1 strains with specific genotypes suggests that they have the ability to cause infections in humans [28, 29, 14]. Among the *L. pneumophila* sg 1 strains, some monoclonal subgroups (Knoxville, Philadelphia, Benidorm, France/Allentown) display the virulence-associated epitope recognised by MAb 3/1; these strains seem to be associated with hospital outbreaks, travel-associated infections or community-acquired infections [26, 10]. There are some published data [10, 13, 14, 30, 31] on the serogroup and monoclonal subgroup distribution of *L. pneumophila* in man-made water systems that are associated or not associated with human disease. This paper describes the first Italian study in which information about the environmental distribution of strains according to their MAb 3/1 profile has been examined. This distribution was compared with the occurrence of legionellosis.

Our laboratory is involved in the environmental monitoring of many HCFs in Piemonte (area 25,399 km^2^; population 4,446,230); thus, we collected environmental strains from water systems in HCFs that are representative of the wider *L. pneumophila* population in our region.

The purpose of this study was to compare the distribution of *L. pneumophila* sg 1 monoclonal subtypes obtained during routine sampling in 56 man-made HCF water systems with the distribution of hospital-acquired legionellosis, to assess the risk associated with contaminated environmental reservoirs colonised by more virulent strains.

We could acknowledge two methodological limitation of this study: first, owing to this investigation being an ecological study, we didn’t observe the characteristics of the patients involved and we cannot exclude that we could not identify and consider some potential confounding variables; second, since diagnosis of legionellosis in Piemonte is based on urinary antigen detection, it is not possible an epidemiological comparison between clinical and environmental strain to confirm the infection origin.

Of the 56 health care facilities that we monitored, 26 were colonised by *L. pneumophila* sg 1 alone or in association with other *L. pneumophila* serogroups. Only 8 (31%) of these facilities were colonised by MAb 3/1-positive *L. pneumophila* sg 1, indicating that the total frequency of colonisation by MAb 3/1-positive *L. pneumophila* sg 1 was 14% (8/56) among the HCFs. This observation aligns with the findings of other studies that most environmental isolates are MAb 3/1-negative, in contrast to human isolates [10, 32–34]. The comparison between the environmental data and the data on the cases of Legionnaires’ disease showed that 93% of the cases (81/87) occurred in the HCFs colonised by *L. pneumophila* sg 1 alone or with others serogroups. Among the *L. pneumophila* sg 1 strains, the monoclonal subgroup with the virulence-associated epitope recognised by MAb 3/1 was isolated in 27% (7/26) of the hospitals, which had reported 85% (74/87) of the cases (Table 1). Only one health care facility colonised by MAb 3/1-positive *L. pneumophila* sg 1 (No. 9, Table 1) had no reported cases of legionellosis. From 2004 to 2006 this hospital had two medical wards (64 beds) while from 2005 to 2009 was no longer an hospital but was intended only for hemodialysis center. Moreover in this hospital was highlighted a variation of the reactivity of MAb 3/1 from positive to negative.

Only one health care facility colonised by MAb 3/1-negative *L. pneumophila* sg 1 (No. 6, Table 1) reported four cases of legionellosis. Despite the epidemological investigation, the infection sources remained unknown. Health authorities hypothesized the involvement of the cooling towers of a supermarket in the nearby of the hospital.

The epidemiological data for Piemonte (Table 1) show that the reporting rates are particularly high at hospitals that admit patients with severe illnesses; therefore, we analysed the relationship between CMI, MAb and hospital-acquired infections in the 56 HCFs. As shown in Table 5, we found a statistically significant relationship between the presence of strains MAb 3/1 positive in the environment and the occurrence of cases of legionellosis. After stratification for CMI, the association was confirmed only for low-complexity hospitals (CMI < 1) while disappeared for high complexity hospitals (CMI > 1). These results might suggest that the subjects with less severe illnesses may need more virulent strains to get sick while patient with severe underlying disease, hospitalised in facilities with CMI > 1, were more susceptible to becoming infected even by less aggressive strains. This observation aligns with the findings of Helbig and colleagues [10], who found that most clinical isolates in hospitals are MAb 3/1-negative.

## Conclusions

Our study suggests that MAb 3/1 subtyping of *L. pneumophila* sg 1 strains may be useful for assessing the risk associated with contaminated environmental reservoirs. Hospitals colonised by more virulent strains should be advised to increase their case monitoring efforts and to implement more effective contamination control strategies. Many of the guidelines and official recommendations for preventing the transmission of Legionnaires’ disease recommend action items based solely on certain concentrations of legionellae or on the number of sites that are positive for the bacteria [35, 36]. None of these guidelines discriminate among the large number of *Legionella* species or serogroups, even though it is clear that they differ in their capacity to cause disease. Better characterisation of the strains that cause the majority of diseases would allow for more targeted intervention measures.

Our results support the opinion stated by Harrison et al. [14]. That “knowing which particular strain is present in an environment might be at least as important as knowing the quantity in which legionellae are present”.
